# Curcumin analogue BDDD-721 exhibits more potent anticancer effects than curcumin on medulloblastoma by targeting Shh/Gli1 signaling pathway

**DOI:** 10.18632/aging.204161

**Published:** 2022-07-06

**Authors:** Weiyi Gong, Wenxuan Zhao, Gang Liu, Lei Shi, Xia Zhao

**Affiliations:** 1 Department of Neurosurgery, Gusu School, Nanjing Medical University, The First People’s Hospital of Kunshan, Suzhou 215300, China; 2 Department of Gastrointestinal Surgery, Gusu School, Nanjing Medical University, The First People’s Hospital of Kunshan, Suzhou 215300, China; 3 Department of Oncology, The Yancheng Clinical College of Xuzhou Medical University, The First People’s Hospital of Yancheng, Yancheng 224006, China

**Keywords:** BDDD-721, curcumin, hedgehog pathway, medulloblastoma

## Abstract

Medulloblastoma (MB) is a malignant tumor in the fourth ventricle of children. The clinical treatment is mainly surgical resection combined with radiotherapy and chemotherapy, but the curative effect is not ideal, and the 3-year survival rate is very low. Previous study confirmed that curcumin attenuated the proliferation of medulloblastoma both *in vitro* and *in vivo*. In present study, we found a curcumin analogue named BDDD-721, exhibited more potent anti-tumor activity than curcumin. Compared with curcumin, BDDD-721 more effectively inhibited the proliferation, migration, invasion, and increased apoptosis of medulloblastoma cells. Furthermore, BDDD-721 treatment led to activation of glioma-associated oncogene homolog (Gli), reduced expression of Shh and its downstream target Smo, Gli1 and Ptch1. In addition, SAG (Shh signaling pathway agonist) antagonized the pro-apoptotic effects of BDDD-721 on medulloblastomas as confirmed by CCK8 assays and flow cytometry; while cyclopamine (Shh signaling pathway inhibitor) enhanced its effects on medulloblastomas. In conclusion, these results indicate that curcumin analogue BDDD-721 has more potent anticancer effects than curcumin on medulloblastomas by targeting Shh/Gli1 signaling pathway.

## INTRODUCTION

Medulloblastoma (MB) is a malignant tumor in the fourth ventricle of children, accounting for 10% ~ 20% of children’s central nervous system tumors [1]. The peak of incidence is about 7 years old. At present, surgical resection is the first choice, followed by radiotherapy and chemotherapy [2]. Medulloblastoma is very sensitive to radiotherapy. Postoperative whole brain and whole spinal cord radiotherapy is a routine adjuvant treatment. The overall survival rate of patients with existing treatment schemes can reach 70% [3]. However, excessive radiotherapy and chemotherapy cause damage to children’s hearing, cognition, endocrine and vascular system, and the long-term survival rate of tumor is still poor [4]. However, its pathogenesis is not very clear. At present, it is considered that the most important carcinogenic pathways of medulloblastomas are involved in sonic hedgehog (Shh), Wnt/β-catenin, Akt/nuclear factor _κ_B (NF-_κ_B), EGFR and other signaling pathways [5].

Curcumin is a polyphenol compound with symmetrical molecular structure consisting of a β-diketone heptadiene linked to two o-methylated phenols [6]. Curcumin from natural sources is distributed in curcuma longa, curcuma zedoaria, curcuma aromatica and other curcuma plants [7]. In recent years, curcumin has attracted extensive attention because of its anti-inflammatory, anti-oxidant, anti-bacterial, anti-fibrosis, anti-angiogenesis and anti-Alzheimer’s disease [8]. Among them, the most studied is its antitumor activity. Its broad-spectrum antitumor activity has been confirmed in a variety of animal experiments, and its antitumor mechanism has become a hot spot in recent research [9]. Recently, curcumin has been found to have inhibited effects on medulloblastoma [10]. However, curcumin has poor water solubility, unstable structure and easy degradation *in vivo*, resulting in its low bioavailability and poor activity in many anti-tumor experiments *in vivo* [11]. Considering these shortcomings, we modified the structure of curcumin and screened curcumin derivatives that were more effective against medulloblastomas. (1E,6E)-1-(4-(diethylamino)phenyl)-7-(4-hydroxy-3-methoxyphenyl)hepta-1,6-diene-3,5-dione named BDDD-721 was found by us to be the most effective compound against medulloblastomas.

In this study, we analyzed the function of BDDD-721 in regulating proliferation, apoptosis, and invasion of medulloblastomas *in vitro* and *in vivo*. And our results confirmed the potential ability of BDDD-721 for therapeutic applications on medulloblastoma via Shh/Gli1 signaling pathway.

## RESULTS

### BDDD-721 has a better suppressive effect on medulloblastoma cell proliferation than curcumin *in vitro*

**Figure 1:**
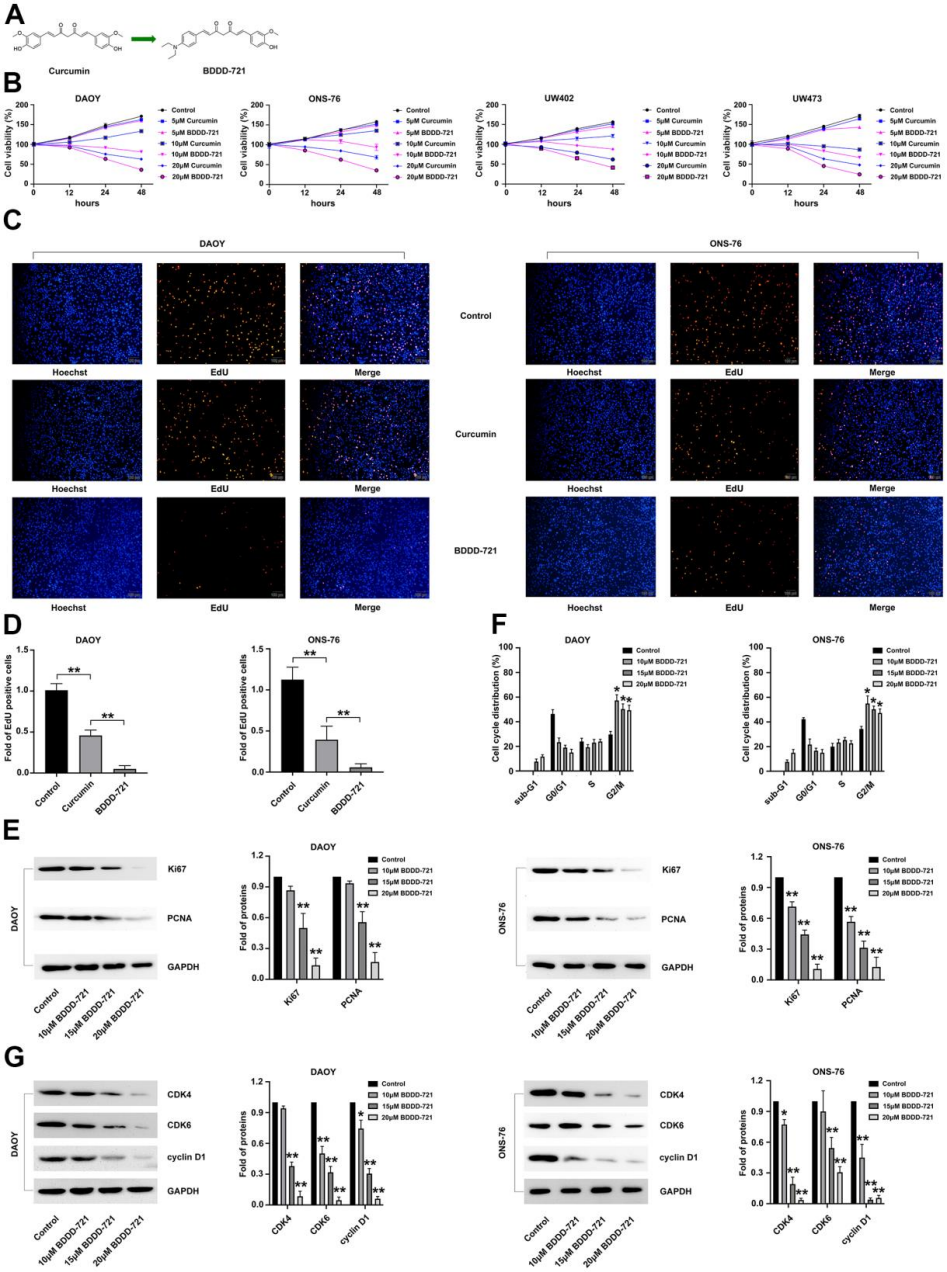
**Effects of BDDD-721 and curcumin on medulloblastoma cells.** (**A**) Chemical structures of BDDD-721. (**B**) Effects of BDDD-721 and curcumin on DAOY, UW402, UW473 and ONS-76 cells were analyzed by MTT assay *in vitro*. (**C**) Effects of BDDD-721 and curcumin on cell viability were evaluated by EdU assay. (**D**) Column chart represents the percentage of EdU positive cells analyzed by SPSS statistical software. (**E**) The proliferation antigen Ki67 and PCNA were detected by western blot. (**F**) Cell cycle was analyzed by flow cytometry following propidium iodide staining for DNA content after BDDD-721 treatment for 24h. (**G**) Cell cycle associated proteins of Cyclin D1, CDK4, and CDK6 were examined by western blot. Compared with Control, *p < 0.05, **p < 0.01.

In present study, we investigated the cytotoxic effects of curcumin and BDDD-721 against medulloblastoma cells by using MTT assay (Figure 1A). As shown in Figure 1B, DAOY, UW402, UW473 and ONS-76 cells were treated with different concentrations of curcumin and BDDD-721 for 48 h, and data showed dose-dependent effects of curcumin and BDDD-721 on medulloblastoma cells. Moreover, either curcumin or BDDD-721 over 10 μM exhibited significantly inhibitory effects on medulloblastoma cell growth after 24 h.

Then, EdU assay was further applied to detect the proliferation of DAOY and ONS-76 cells after 20 μM curcumin and BDDD-721 treatment. As shown in Figure 1C, either 20 μM curcumin or BDDD-721 could reduce the percentage of EdU positive cells; however, more EdU positive cells were found in BDDD-721 than curcumin treatment group (P<0.05) (Figure 1D). Simultaneously, western blot results demonstrated that medulloblastoma cell associated proliferation antigen Ki67 and PCNA were significantly decreased after 20 μM BDDD-721 treatment for 24 h (Figure 1E). Taken together, BDDD-721 has a better suppressive effect on medulloblastoma cell proliferation than curcumin.

Cell proliferation is often accompanied by cell cycle arrest. We further explored whether BDDD-721 induced cell cycle arrest in human DAOY and ONS-76 cells. DAOY and ONS-76 cells were further exposed to 10, 15, 20 μM concentrations of BDDD-721 for 12 h and then cell cycle was analyzed by flow cytometry. As shown in Figure 1F, cell G2/M phase arrest was shown in BDDD-721-treated DAOY and ONS-76 cells. Moreover, the levels of cell cycle associated proteins Cyclin D1, CDK4, and CDK6 were detected by western blot analysis and showed markedly reduced in the BDDD-721-treated DAOY and ONS-76 cells (Figure 1G). These results suggested that BDDD-721-induced proliferation inhibition was involved in G2/M phase cell cycle arrest in human medulloblastomas.

### BDDD-721 induces more medulloblastoma cell apoptosis than curcumin *in vitro*

**Figure 2:**
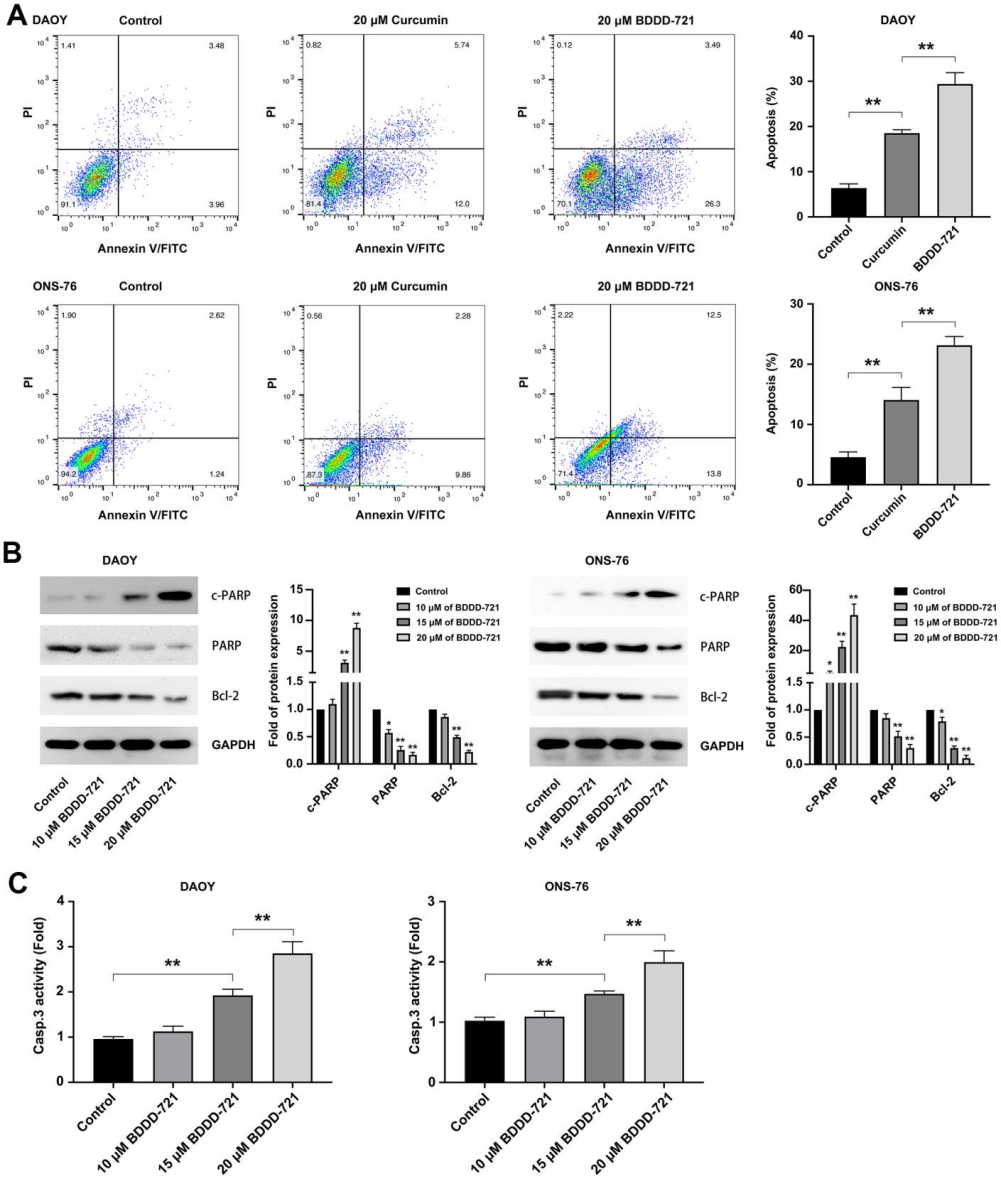
**BDDD-72 and curcumin triggered apoptosis against medulloblastoma cells.** (**A**) Cell apoptosis was examined by flow cytometry assay using the Annexin V/PI after BDDD-721 or curcumin treatment for 24h. (**B**) Apoptosis-related proteins of cleaved PARP, PARP and Bcl-2 were analyzed by western blot. (**C**) Caspase-3 activity was measured by ELISA assay. Compared with Control, *p < 0.05, **p < 0.01.

To compare the apoptotic effects of BDDD-721 and curcumin on medulloblastoma cells, DAOY and ONS-76 cells were incubated with 20 μM BDDD-721 and curcumin for 24 h, and the rate of apoptosis was further evaluated by Annexin V/PI assay. As shown in Figure 2A, 20 μM BDDD-721 induced (29.40±2.50)% and (23.13±1.46)% apoptosis of DAOY and ONS-76 cells, respectively. Meanwhile, 20 μM curcumin induced apoptosis of DAOY and ONS-76 cells by (18.53±0.75)% and (14.07±2.07)%, respectively. These data suggested compared with curcumin, BDDD-721 has a stronger ability to induce apoptosis of medulloblastoma cells (P<0.05). Further, the extrinsic apoptosis pathways including PARP, and Bcl-2 were examined by western blot (Figure 2B), and activation of caspase-3 was analyzed by ELISA assay at 12h after treatment (Figure 2C). The results showed the activation of caspases by BDDD-721 treatment, followed by the activation of caspase-3, PARP, and degradation of Bcl-2. These results indicated that BDDD-721 induced apoptosis of medulloblastomas was involved in activation of caspases apoptotic pathway.

### Compared with curcumin, BDDD-721 exerts a better repressive effect in medulloblastoma cell migration and invasion

**Figure 3:**
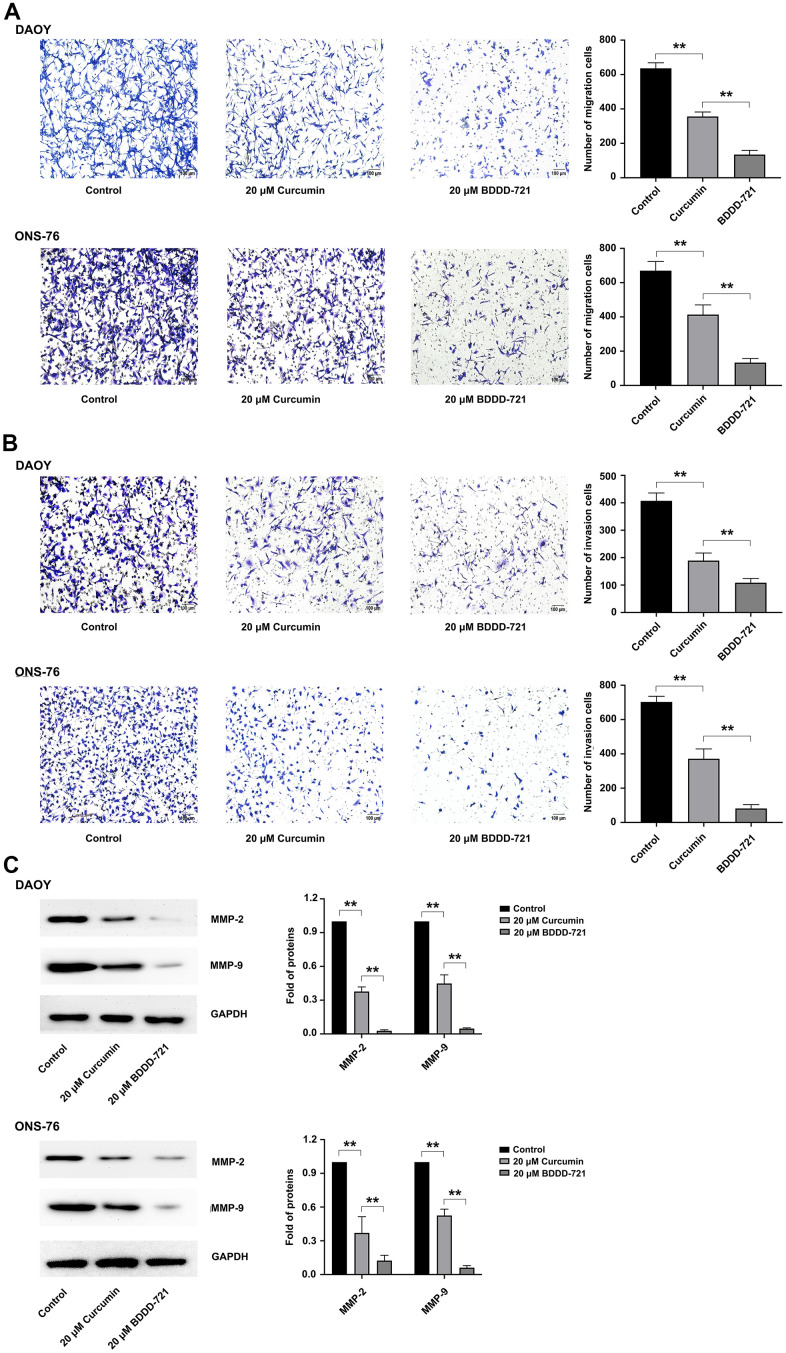
**Effects of BDDD-721 and curcumin on cell migration and invasion of medulloblastoma cells.** (**A**) Cell migration ability was analyzed by Transwell assay without coated Matrigel. (**B**) Cell invasion ability was measured by Transwell assay with coated with Matrigel. (**C**) MMP-2 and MMP-9 proteins were detected by western blot after 20 μM curcumin or BDDD-721 treatment for 24h.

Both DAOY and ONS-76 represent highly invasive characteristics *in vitro* and *in vivo* [12]. In accordance with this, the effects of curcumin and BDDD-721 on medulloblastoma cell migration and invasion were then assessed. In the migration assay, with regard to curcumin-treated cells, BDDD-721 showed a more significant ability to inhibit cell migration (Figure 3A). In the transwell assay, as shown in Figure 3B, it was clear that fewer cells of DAOY and ONS-76 migrated to the lower surface in BDDD-721 treatment group compared with curcumin. Furthermore, proteins closely associated with cell invasion and migration were detected. As shown in Figure 3C, MMP-2 and MMP-9 were detected after 20 μM curcumin or BDDD-721 treatment by western blot analysis. Our data showed that either curcumin or BDDD-721 decreased the expression MMP-2 and MMP-9 proteins, which was lower in BDDD-721 treatment group. Therefore, compared with curcumin, BDDD-721 exerted a better repressive effect in medulloblastoma cell migration and invasion.

### BDDD-721 inactivates the hedgehog pathway in medulloblastoma cells

**Figure 4:**
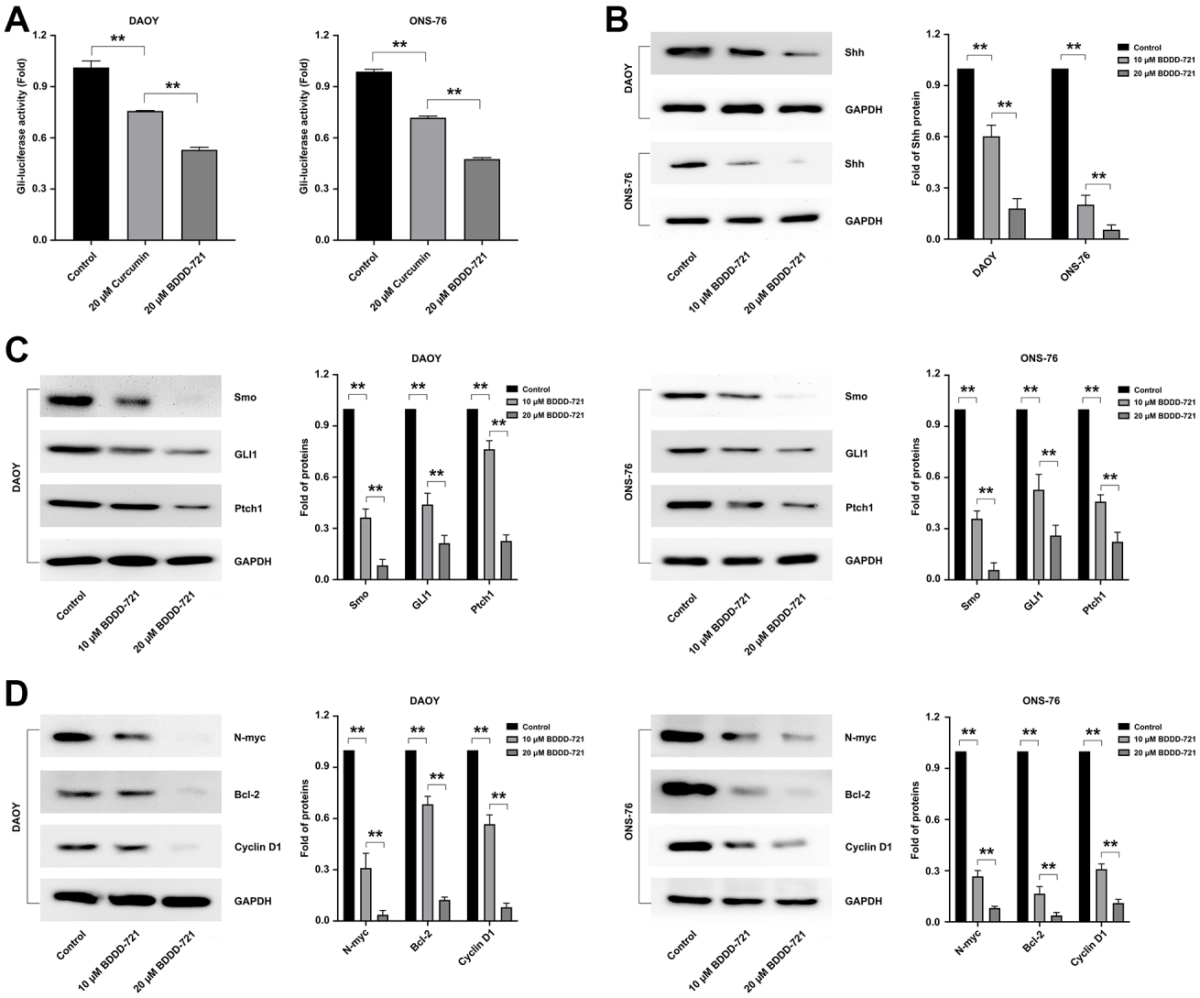
**Effects of BDDD-721 on the hedgehog pathway in medulloblastoma cells.** (**A**) Gli activity was detected by Gli-luciferase reporter assay in medulloblastoma cells treated with curcumin or BDDD-721. (**B**) The expression of Shh protein was analyzed by western blot in DAOY and ONS-76 cells treated with BDDD-721 for 48h. (**C**) Proteins of Smo, GLI1 and Ptch1 were examined by western blot in DAOY and ONS-76 cells treated with BDDD-721 for 48h. (**D**) Proteins of N-myc, Bcl-2 and Cyclin D1 were quantified by western blot in DAOY and ONS-76 cells treated with BDDD-721 for 48h.

Recently, aberrantly activated hedgehog signaling pathway has been found in many cancers including medulloblastomas, which was also involved in antitumor mechanism of curcumin [13]. To identify whether BDDD-721 also regulated the hedgehog signaling, Gli-luciferase reporter assay was conducted. According to Figure 4A, the activation of Gli-luciferase reporter in medulloblastoma cells could be reduced by 20 μM curcumin or BDDD-721, and BDDD-721 led to a larger fold change. Further, DAOY and ONS-76 cells were treated with BDDD-721 (0, 10, 20 μM) for 48 h, and the results showed that Shh protein (Figure 4B) and its downstream targets including Smoothened (Smo), glioma-associated oncogene homolog 1 (Gli1) and patched homolog 1 (Ptch1) significantly decreased with increasing concentrations of BDDD-721 (Figure 4C); To further elucidate the effect of BDDD-721 on the hedgehog pathway, the main Shh and Gli1 downstream targets of N-myc, Bcl-2 and Cyclin D1 were detected by western blot. As shown in Figure 4D, a sharp decrease of these proteins was found in DAOY and ONS-76 cells after BDDD-721 treatment for 48 h. These data demonstrated that BDDD-721 inhibited the Shh/Gli1 signaling in medulloblastoma cells.

### Shh signaling is required for BDDD-721 inhibition of cell growth and induction of apoptosis in medulloblastomas

**Figure 5:**
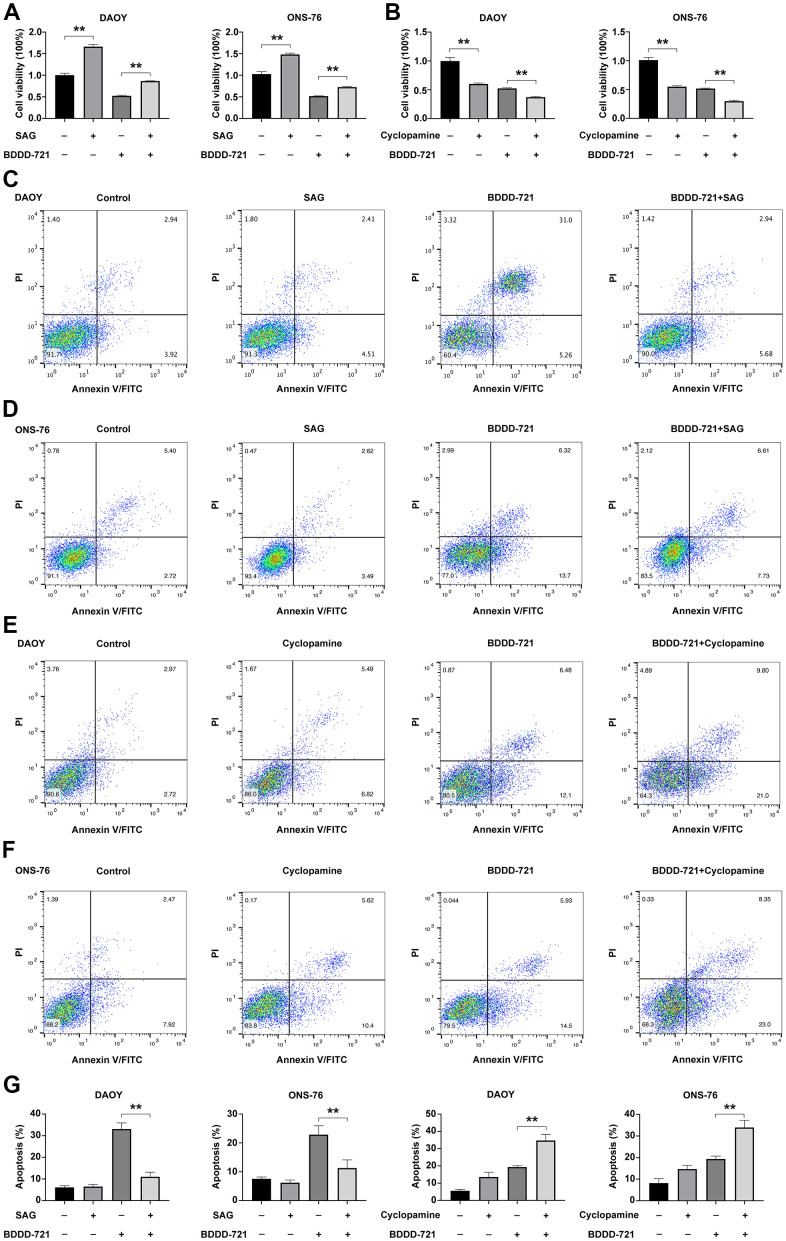
**Hedgehog signaling is required for BDDD-721 against medulloblastoma cells.** (**A**) The cell viability of DAOY and ONS-76 cells were analyzed by MTT assay in DAOY and ONS-76 cells treated with BDDD-721 and/or SAG. (**B**) The cell viability of DAOY and ONS-76 cells were analyzed by MTT assay in DAOY and ONS-76 cells treated with BDDD-721 and/or cyclopamine. Cell apoptosis was detected by flow cytometry in DAOY (**C**) and ONS-76 (**D**) cells treated with BDDD-721 and/or SAG. Then, cell apoptosis was detected by flow cytometry in DAOY (**E**) and ONS-76 (**F**) cells treated with BDDD-721 and/or cyclopamine. (**G**) Column chart represents the percentage of apoptosis cells analyzed by SPSS statistical software.

To further examine whether Shh signaling was required for BDDD-721 inhibition of medulloblastoma cell growth and induction of its apoptosis, DAOY and ONS-76 cells were treated with 10 μM cyclopamine (Shh signaling specific inhibitor), BDDD-721, and/or 0.6 μM SAG (Shh signaling specific agonist) for 48 h. As shown in Figure 5A, cell viability was analyzed by MTT assay, and showed 0.6 μM SAG increased cell viability of DAOY and ONS-76 cells, while 20 μM BDDD-721 significantly decreased cell viability of DAOY and ONS-76 cells. However, combination of 0.6 μM SAG and 20 μM BDDD-721 partially rescued BDDD-721-induced inhibition of medulloblastoma cell growth in both DAOY and ONS-76 cells. In contrast, when cells treated with Shh signaling specific inhibitor cyclopamine, 10 μM cyclopamine decreased cell viability of DAOY and ONS-76 cells to 60.21% and 54.37%, respectively. However, a combination of 10 μM cyclopamine and 20 μM BDDD-721 significantly augmented BDDD-721-induced cell inhibition of DAOY and ONS-76 cells (Figure 5B). Then, cell apoptosis was analyzed by flow cytometry. Compared with control group, 0.6 μM SAG did not increase any apoptosis rate in DAOY and ONS-76 cells, while 20 μM BDDD-721 significantly increased the cell apoptosis of DAOY and ONS-76 cells (Figure 5C, 5D, 5G). Nevertheless, combination of 0.6 μM SAG and 20 μM BDDD-721 rescued BDDD-721-induced cell apoptosis of DAOY and ONS-76 cells. We also treated DAOY and ONS-76 cells with 10 μM cyclopamine, and showed increased apoptosis in Figure 5E–5G. Moreover, more significant apoptosis was further found in the combined treatment group of 10 μM cyclopamine and 20 μM BDDD-721. These results indicated that the effects of BDDD-721 on anti-medulloblastomas were involved in Shh signaling pathway.

### Comparison of curcumin and BDDD-721 on the anti-tumor effects of medulloblastoma *in vivo*

**Figure 6:**
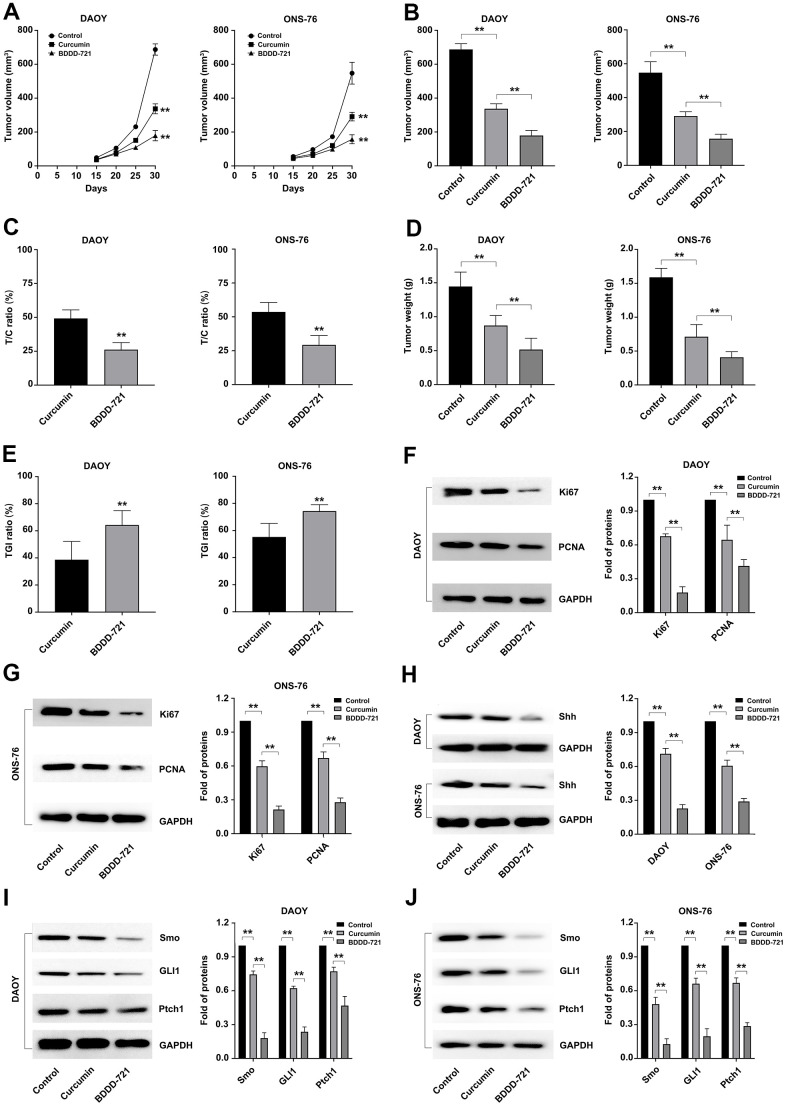
**Effects of curcumin and BDDD-721 against medulloblastoma cells *in vivo*.** (**A**) The tumor volume of xenografts was recorded after curcumin and BDDD-721 treatment. (**B**) The difference of the final median tumor volume between curcumin and BDDD-721 treatment was analyzed by SPSS statistical software. (**C**) T/C (%) was calculated and analyzed after curcumin and BDDD-721 treatment. (**D**) The difference of the final weight of transplanted tumor between curcumin and BDDD-721 treatment was analyzed by SPSS statistical software. (**E**) TGI(%) was calculated and analyzed after curcumin and BDDD-721 treatment. Then, the expression of Ki67 and PCNA proteins were detected by western blot in DAOY (**F**) and ONS-76 (**G**) xenografts. (**H**) Shh protein was detected by western blot in DAOY and ONS-76 xenografts. And the expression of Smo, GLI1 and Ptch1 proteins were detected by western blot in DAOY (**I**) and ONS-76 (**J**) xenografts.

To further analyze the anti-tumor effects of BDDD-721 *in vivo*, subcutaneous transplantation tumor xenografts of DAOY and ONS-76 cells were conducted in the flank regions of the nude mouse. To compare the chemopreventive effect of curcumin and BDDD-721 on tumorigenesis, curcumin and BDDD-721 were administered via an intraperitoneal injection and started at 3 days after tumor cell inoculation. Our preliminary results of mouse intraperitoneal LD50 of BDDD-721 was 876.38 mg / kg. And according to previously reported literature, the recommended dose for curcumin *in vivo* experiment was 60 mg / kg. Thus, 60 mg / kg was selected to carried out the treatment in this study. As shown in Figure 6A, both of curcumin and BDDD-721 could inhibit the growth of xenografts; however, BDDD-721-treated mice exhibited significantly smaller tumors than curcumin-treated mice after the 25th day. And according the final median tumor volume (Figure 6B), relative tumor proliferation rate T/C (%) of BDDD-721 was also smaller than that in curcumin-treated group at the end of the experiment, 26.20% vs. 49.25% in DAOY cells and 29.28% vs. 53.64% in ONS-76 cells, separately (Figure 6C). And as expected, the weight of transplanted tumor of BDDD-721-treated group (0.52±0.17g) was also lower than that of curcumin-treated group (0.87±0.15g) (Figure 6D). As shown in Figure 6E, according to the data of the transplanted weight, the TGI(%) in BDDD-721-treated group vs. curcumin-treated group were 64.27% vs. 38.69% in DAOY cells and 74.32% vs. 55.23% in ONS-76 cells, separately; the former was significantly higher than the latter (P<0.05). Overall, these results demonstrated that BDDD-721 was able to significantly inhibit the growth of medulloblastoma xenografts *in vivo*.

Further, the medulloblastoma related proliferation marker proteins of Ki67 and PCNA were detected in xenografts by western blot, and results showed more significantly reduced proteins of Ki67 and PCNA in BDDD-721 than that in curcumin-treated group (Figure 6F, 6G). Moreover, western blot analysis further confirmed the levels of Shh/Gli signaling proteins including Shh and its downstream Smo, Gli1 and Ptch1. As shown in Figure 6H–6J, compared with the control group, BDDD-721 and curcumin could inhibit the expression of Shh, Smo, Gli1 and Ptch1 proteins in transplanted tumors to a certain extent, and the effect of BDDD-721 was stronger (P<0.05). These results suggested that BDDD-721 had more obvious inhibitory efficiency on the growth of medulloblastoma xenografts.

## DISCUSSION

Medulloblastoma is one of the most common central nervous system tumors in children [12]. At present, the clinical treatment for medulloblastoma is mainly surgery combined with radiotherapy and chemotherapy [13]. According to statistics, these treatments can cause long-term side effects, such as neurocognitive deficits, hormone deficiency, and serious ototoxicity caused by platinum drugs, leukemia, etc. [14]. It has been reported that curcumin has a strong inhibitory effect on medulloblastoma cells and basically has no toxic side effects. However, due to the low water solubility and poor stability of curcumin, the disadvantages of large dosage required to exert its medicinal effect hinder its clinical application and promotion [15].

During present study, in order to overcome the above shortcomings of curcumin, we synthesized a variety of curcumin derivatives and selected a curcumin derivative BDDD-721 that was more effective in the treatment of medulloblastoma. We compared the inhibited effects of curcumin and its derivative BDDD-721 on medulloblastoma cell lines both *in vitro* and *in vivo*, and data showed BDDD-721 had more potent anticancer effects against medulloblastoma either *in vitro* or *in vivo*. Consider the presence of tumor heterogeneity, four different medulloblastoma cell lines of DAOY, UW402, UW473 and ONS-76 were conducted in this study. DAOY, UW402 and UW473 were classified into TP53 mutant Sonic Hedgehog (Shh) subgroup and characterized by high migration potential; while ONS-76 was grouped in the TP53 wildtype Shh subgroup and characterized by high proliferative potential [16]. However, only DAOY and ONS-76 cell lines had the tumorigenic potential *in vivo* [17]. Herein, the *in vitro* anticancer activity of BDDD-721 has been achieved on these four different cell lines by MTT assay, and data showed BDDD-721 had more potent inhibited effects on all the cells than curcumin. Further EdU experiments also confirmed this on DAOY and ONS-76. Moreover, cell proliferation-related indicators Ki67 and PCNA also showed a more significant decrease in BDDD-721-treated group than that in curcumin-treated group. To further characterize the effects of BDDD-721 as a more potent chemotherapeutic agent for medulloblastoma, we decided to compare its effects to curcumin *in vivo*. Compared with the curcumin-treated group in the medulloblastoma cell xenograft model, BDDD-721 treatment more significantly reduced tumor volume and mass. Protein analysis also showed similar results that treatment with BDDD-721 and curcumin reduced the proliferation Ki67 and PCNA of xenograft tumor, and the inhibition effect of BDDD-721 was stronger at the same dose.

The phenomenon of cell proliferation inhibition was also reflected in the change of cell cycle, and the cells intervened by BDDD-721 showed obvious G2/M phase arrest. Activated Cyclin D1/CDK4 and Cyclin D1/CDK6 complex were essential for S phase entry [18]. Curcumin was reported to inhibits mammary epithelial carcinoma cell cycle progression by reducing Cyclin D1 expression and blocking its associated CDK4/CDK6 proteins [19]. In present study, we first detected the expression of CDK4, CDK6 and Cyclin D1 and showed BDDD-721 significantly reduced CDK4, CDK6 and Cyclin D1 expression. According to flow cytometry analysis, with the increase of drug dose, the Sub-G1 peak appeared before the G0/G1 peak, indicating that the cells undergo spontaneous apoptosis [10]. In this study, we found that curcumin could promote apoptosis in medulloblastoma cells, which was consistent with previous studies [10], but the effect of BDDD-721 was more pronounced. And as seen from the changes of increased cleaved PARP, activity of caspase-3 and decreased Bcl-2 expression with the increased dose of BDDD-721, BDDD-721 activated the mitochondria apoptotic pathways in medulloblastoma cells. In addition, cell migration and invasion ability were further confirmed to be significantly inhibited by BDDD-721 superior than curcumin, and the cell migration and invasion-associated MMPs molecules of MMP-2 and MMP-9 were also decreased. These results suggested BDDD-721 had a stronger effect than curcumin on medulloblastoma cells in terms of cell proliferation, cycle, migration, and invasion, which indicated that BDDD-721 had a more effective therapeutic effect on medulloblastoma cells.

In recent years, the mechanisms of curcumin in different tumors were found to be not consistent. Khaw et al. found that curcumin inhibited the growth of glioblastoma and medulloblastoma cells by shortening the telomere [20]. Bangaru et al. showed curcumin had a potent anti-proliferative effect on medulloblastoma cells mediated by NF-κB suppression [21]. However, Elamin et al. confirmed that curcumin triggered apoptosis of medulloblastoma cells by suppressing the Shh-Gli1 signaling pathway [22]. In addition, He et al. explored that Wnt/β-catenin was the mechanism by which curcumin inhibited medulloblastomas [23]. But the underline mechanism of the curcumin analog BDDD-721 was still unclear. In this study, we found BDDD-721 could decrease the Gli1 and Bcl-2 expression. And Bcl-2 is one of the direct targets of Shh/Gli1 signaling [24]. Thus, we hypothesized that Shh/Gli1 signaling pathway was involved in the anti-medulloblastoma mechanism of BDDD-721. As expected, we first checked the Shh expression and confirmed Shh was significantly inhibited by BDDD-721. Shh/Gli1 signaling is activated on tumorigenicity of cancer cells [25]. Shh induces the transcription of two important transcription factors Gli1 and Ptch1 in many cell types. Canonical activation of Shh signaling occurs through binding of its ligands to Ptch1, which derepresses the transmembrane G protein-coupled receptor SMO. And Gli1, the terminal effectors of Shh signaling, are released from suppressor of fused-mediated cytoplasmic sequestration, permitting nuclear translocation and activation of target genes, such as N-myc, Cyclin D1, Foxm1, and Bcl-2 [26]. Our further data showed Smo, Gli1 and Ptch1 were downregulated in a dose-dependent manner after BDDD-721 treatment, and several Gli1-dependent target genes of Cyclin D1, Bcl-2 and N-myc were also downregulated. Then, we confirmed that the BDDD-721-induced growth inhibition and apoptosis could be blocked by SAG, a Shh signaling specific agonist and enhanced by cyclopamine, a Shh signaling specific antagonist. These data suggested that inactivation of Shh/Gli1 signaling was involved in BDDD-721-induced growth inhibition and apoptosis of medulloblastoma cells.

In conclusion, these data suggest that BDDD-721 effectively inhibits proliferation, induces their apoptosis and alleviates the migration and invasion of medulloblastoma cells. In addition, our data demonstrate that the underlying mechanism is through inhibiting Shh/Gli1 signaling pathway. These findings indicate that BDDD-721 is superior to curcumin in medulloblastoma treatment. Therefore, BDDD-721 may be a promising drug for preventing and treating the progression of medulloblastomas.

## MATERIALS AND METHODS

### Cell lines and culturing conditions

Four medulloblastoma cell lines of DAOY, UW402, UW473 and ONS-76 were used in this study. DAOY cell line was cultured in a-MEM medium containing 10% fetal bovine serum (FBS; Gibco, Grand Island, NY, USA). ONS-76 cell line was cultured in RPMI-1640 medium containing 10% FBS (Grand Island, NY, USA). UW402 and UW473 cell lines were cultured in Dulbecco’s modified Eagle’s medium (DMEM)-F12 medium with 10% FBS (Grand Island, NY, USA) in an incubator at 37° C and 5% CO_2_. All culture medium contained 100 U/ml penicillin and 100 mg/ml streptomycin (Grand Island, NY, USA). All cells were grown a humidified atmosphere of 5% CO_2_ at 37° C.

### BDDD-721 synthesis

In this study, a novel analog of curcumin bearing N atom was designed and prepared to improve the drug likeness of curcumin following the synthetic protocol for curcumin. Briefly, boric anhydride was first reacted with acetylacetone to generate an acetone-boric oxide complex to prevent side reaction, and subsequent single aldol condensation with 4-diethylaminobenzaldehyde afforded the intermediate. Finally, this intermediate subjected to a second aldol condensation with vanillin afford the target compound BDDD-721. The structure of synthesized BDDD-721 was fully characterized by nuclear magnetic resonance (NMR) and mass spectroscopy. The purity of BDDD-721 was ≥ 95%, as estimated by HPLC.

### Cytotoxicity assay

Logarithmic phase cells were seeded in 96-well plates with a density of 1×10^4^ cells/well and maintained under standard culture conditions for 24 h. Then cells were treated with indicated concentrations of curcumin and BDDD-721 for 48 h. 50 μL MTT (Genbio, Suzhou, China) solution was added to each well, and the culture was continued for 4 h. Aspirate the supernatant, add 150 μL of DMSO (Sigma-Aldrich, St. Louis, MO, USA) to each well to dissolve the formazan, and shake well with a plate shaker. The absorbance at 490 nm was read using an iMax Microplate Reader (BioTek® Instruments, Inc., Winooski, Vermont, USA) and the results were recorded. The cell growth curve was drawn with the time as the abscissa and the absorbance value as the ordinate.

### EdU assay

Cells in logarithmic growth phase were taken and seeded in 96-well plates at 1×10^4^ cells/well, and cultured to normal growth stage. Then cells were treated with indicated concentrations of curcumin and BDDD-721 for 24 h. An EdU assay kit obtained from Genbio (Suzhou, China) was used to determine cell growth according to the manufacturer’s manual. Briefly, cells were incubated with 100 μM of EdU buffer at 37° C for 2 h, fixed with 50μL cell fixative for 30 min and dyed with Dyeing reaction solution for 30 min. Finally, each well was added 100 μL of 1X Hoechst 33342 reaction solution and incubated for 30 min. Then, cell nuclei was observed and recorded by using a fluorescence microscope (Nikon, Japan). Quantitative data were expressed as a percentage of EdU-positive nuclei relative to the total number of nuclei counted.

### Transwell for migration and invasion assay

Cells were pre-treated with or without 20 μM curcumin or BDDD721 for 4 h. Then, 1 × 10^5^ cells in serum-free media were placed into the upper chamber of an 8-μm Transwell filter (BD Biosciences Pharmingen, San Diego, CA, USA) coated with or without 25 μL Matrigel (1:4, 354234, Beckton Dickinson, Franklin Lakes, NJ, USA). Transwell filters coated with Matrigel were used for invasion experiments, while those without coating were used for migration experiments. Then, 600μl of culture media containing 10% FBS was added into the lower chamber. After incubation for 24 h, cells inside the chamber were removed with a cotton swab. Cells on the bottom were fixed with 4% paraformaldehyde for 15 min and stained with 0.1% crystal violet (Sigma-Aldrich, St. Louis, MO, USA). The number of transmembrane cells was photographed by using a fluorescence microscope (Nikon, Japan) and counted in five random fields of view.

### Gli-luciferase reporter assay

To determine Gli activity, Gli-Luc luciferase reporter plasmids were constructed by Yeasen (11524ES03, Shanghai, China). Then plasmids were transfected into cells by using Lipofectamine 3000 reagent (Thermo Fisher Scientific, Waltham, MA, USA). After transfection for 24 h, cells were then treated with curcumin and BDDD-721 for 24 h. Finally, cell lysates were prepared, and the firefly and Renilla luciferase activities were analyzed by Dual-Luciferase Reporter Assay System (Promega, Madison, WI, USA). Firefly luciferase activity was used to normalization of Renilla luciferase activity to control for cell numbers. Data were expressed as fold increase over control group.

### Nude mouse model

Female BALB/c-nu/nu nude mice were provided by the Institute of Laboratory Animals, Changzhou Cavens Experimental Animal Co., Ltd. (Changzhou, China). Nude mice were divided into three groups with 10 mice in each group. Logarithmic growth cells of 5 × 10^6^ cells were suspended in 100 μl serum-free DMEM and injected subcutaneously into the flanks of nude mice. To determine the effect of BDDD-721 and curcumin on subcutaneously xenografted tumors, nude mice received daily intraperitoneal (i.p.) treatment with 60 mg/kg BDDD-721 or curcumin on day 3 after tumor implantation. The tumor volumes were measured by a caliper, and the volume formula was A × B^2^ × 0.5 (A, tumour length; B, width). The tumor weight was measured when the animals were sacrificed. Relative tumor proliferation rates T/C (%) were calculated using the following formula:T_RTV_/C_RTV_*100% (T_RTV_, median tumor volume of treated group on terminal day; C_RTV_, median tumor volume of control group on terminal day). The tumor growth inhibition rates TGI (%) were calculated using the following formula: 1-T_RTW_/C_RTW_*100% (T_RTW,_ treatment group tumor weight on terminal day; C_RTW_, control group tumor weight on terminal day). The animal experiments were approved by the Animal Ethical Protection Association of the First People’s Hospital of Kunshan, and conducted in accordance with the NIH guidelines for the care and use of laboratory animals.

### Western blot analysis

Treated cells were collected and lysed by using RIPA buffer (Sigma-Aldrich, St. Louis, MO, USA). After centrifugation for protein extraction, proteins were separated in 10% SDS-PAGE gels, and then these proteins were further transferred to PVDF membrane (Millipore, Burlington, MA, USA). Then the membranes were incubated overnight at 4° C with primary antibodies of anti-Shh (1:1000, ab53281, Abcam, Cambridge, UK), anti-PARP (1:1000, ab191217, Abcam, Cambridge, UK), anti-Cleaved PARP (1:1000, ab225715, Abcam, Cambridge, UK), anti-Bcl-2(1:1000, ab182858, Abcam, Cambridge, UK), anti-GAPDH (1:1000, ab9485, Abcam, Cambridge, UK), anti-MMP-2 (1:1000, ab92536, Abcam, Cambridge, UK), anti-MMP-9 (1:1000, ab283575, Abcam, Cambridge, UK), anti-Smoothened (1:1000, ab236465, Abcam, Cambridge, UK), anti-Gli1 (1:1000, ab217326, Abcam, Cambridge, UK), anti-Patched / PTCH1 (1:1000, ab53715, Abcam, Cambridge, UK), anti-N-myc (1:1000, ab189528, Abcam, Cambridge, UK), anti-Ki67 (1:1000, ab16667, Abcam, Cambridge, UK), anti-Cyclin D1 (1:1000, ab16663, Abcam, Cambridge, UK), or anti-PCNA (1:1000, ab29, Abcam, Cambridge, UK). After incubation with HRP-conjugated secondary antibodies, the bands were visualized by Chemiluminescent substrate ECL (Genbio, Suzhou, China).

### Statistical analysis

Data were presented as mean ± SD. Differences of groups were analyzed by using a two-tailed unpaired Student’s t-test or two-way ANOVA test (SPSS software, Inc., Chicago, IL, USA). Graphs were generated by using GraphPad Prism 6.0 (GraphPad Software, Inc, San Diego, CA, USA). The level of significance was set at p < 0.05 in all analyses. *, *P* < 0.05 or **, P < 0.01 indicates a statistically significant difference.
